# Exploring Mucin as Adjunct to Phage Therapy

**DOI:** 10.3390/microorganisms9030509

**Published:** 2021-02-28

**Authors:** Amanda Carroll-Portillo, Henry C. Lin

**Affiliations:** 1 Division of Gastroenterology and Hepatology, Department of Internal Medicine, University of New Mexico, Albuquerque, NM 87131, USA; ACarrollPortillo@salud.unm.edu; 2 Medicine Service, New Mexico VA Health Care System, Albuquerque, NM 87108, USA

**Keywords:** phage, mucin, intestine, fecal virome transfer

## Abstract

Conventional phage therapy using bacteriophages (phages) for specific targeting of pathogenic bacteria is not always useful as a therapeutic for gastrointestinal (GI) dysfunction. Complex dysbiotic GI disorders such as small intestinal bowel overgrowth (SIBO), ulcerative colitis (UC), or Crohn’s disease (CD) are even more difficult to treat as these conditions have shifts in multiple populations of bacteria within the microbiome. Such community-level structural changes in the gut microbiota may require an alternative to conventional phage therapy such as fecal virome transfer or a phage cocktail capable of targeting multiple bacterial species. Additionally, manipulation of the GI microenvironment may enhance beneficial bacteria–phage interactions during treatment. Mucin, produced along the entire length of the GI tract to protect the underlying mucosa, is a prominent contributor to the GI microenvironment and may facilitate bacteria–phage interactions in multiple ways, potentially serving as an adjunct during phage therapy. In this review, we will describe what is known about the role of mucin within the GI tract and how its facilitation of bacteria–phage interactions should be considered in any effort directed at optimizing effectiveness of a phage therapy for gastrointestinal dysbiosis.

## 1. Introduction

Bacteriophages (phages) are viruses that infect bacteria. Their discovery in the early 1900s triggered excitement over the prospect of using phages as a therapeutic for treating bacterial infections. While the discovery of antibiotics slowed this line of research in Western societies, Eastern European countries including Russia, Poland, and Georgia continued investigating the use of phages in therapy and prophylaxis, successfully using phages to treat outbreaks of dysentery, typhoid, plague, and cholera [1,2]. Their use of phage therapy focused on several important criteria including harnessing local phages specific to the local bacteria causing any given outbreak, and continuing to create mixtures of phages for cocktails that were more efficient treatments. With the increased prevalence of antibiotic-resistant bacteria and the unwanted side effects of antibiotics in the treatment of gastrointestinal (GI) disorders, focus across the globe has returned to the potential use of phages in anti-microbial treatment. Phage therapy offers an opportunity to use a targeted approach to control the populations of one or more problematic bacteria in order to shift the luminal environment back towards a healthier phenotype. Investigation of the viral component of the microbiome has identified a large, diverse population of phages that are residents of the GI tract [3,4,5,6]. With the presence of an inherent phage population and their likely involvement in regulating numbers of the commensal bacteria comes the hope that manipulating the phage population may lead to an ability to better control the health of the luminal environment. The conventional definition of phage therapy is the treatment of diseases associated with a single pathogen with phages specific to that particular pathogen, and the application of this type of phage therapy has been successful in humans and mice [1,2,7,8,9,10,11]. However, the use of conventional phage therapy for treating all GI disorders has proven to be more elusive. An example of this conundrum is seen in the comparison of phage treatment of *Clostridioides difficile* infections in humans [2,8] versus that of diarrheal disease associated with enterotoxigenic *Escherichia coli* (ETEC; [12,13]). Whereas phage therapy of the former has been successful in humans, clinical studies using coliphage preparations against the latter have not [2,9,14]. Patients afflicted with ETEC-associated diarrhea showed no amelioration of symptoms due to administration of coliphage cocktails [12,13]. It was hypothesized that a lack of effect was likely due to the target of treatment (*E. coli*) occurring in such a small number that coliphage treatment would not have enough bacteria on which to prey and expand. However, disease in these patients was also associated with an increase in the amount of *Streptococcus* in the fecal material suggesting other confounding factors may have played a role. Given these findings, one must consider two possibilities: 1. while ETEC may have been responsible for initiating diarrheal disease, its continued presence was not responsible for sustaining it, and 2. while ETEC causes diarrheal disease, the luminal microenvironment may prevent effective phage–bacterial interaction (supported by the fact that there were still culturable ETEC found within the fecal material). These considerations are particularly pertinent for the concept of phage therapy were targeting a pathogenic organism in a more specific manner than that which occurs with antibiotics is the aim. It is likely that the complexity of the GI microenvironment plays a poorly appreciated but critical role in gastrointestinal dysbiosis and, by default, the overall effect of phage therapy. By modifying the luminal environment, the bacteria–phage interactions necessary to make phage therapy successful may be possible.

Conventional phage therapy of more complex GI disorders—such as small intestinal bacterial overgrowth (SIBO), ulcerative colitis (UC), and Crohn’s Disease (CD)—is not pragmatic as these disorders involve shifts in a number of non-pathogenic, commensal bacterial populations. These disorders are epitomized by the occurrence of a “bloom”, when rare members (such as the sulfate-reducing bacteria *Desulfovibrio*) of the resident bacterial population increase significantly in number, leading to pathologic effects that earn these commensal bacteria the label “pathobiont”. In these instances, preliminary data suggest that a cocktail of multiple phages targeting multiple bacterial species may be more effective [7,8,15,16,17,18,19,20]. However, development of such cocktails is challenging. In instances of human infections, one must rely on bacterial representation in either fecal samples or in vivo sampling that is limited in nature, which may not be indicative of which bacteria to target. Investigation into the spatial distribution of bacteria in the GI system in mice suggests that the length of the intestine is best represented by a compilation of microenvironments with each niche displaying individual properties—something limited sampling would miss [21,22]. Recently, human testing and murine mouse models have been used to investigate an alternative type of phage therapy where an entire “healthy” viral population is transferred into a dysbiotic GI tract (fecal virome transfer or FVT) [17,23,24,25]. The results demonstrate retention of the donor phages over extended periods and amelioration of symptoms associated with *C. dificile* infection [9,14]. While these results are currently limited in scope, this method provides promising opportunity. As these more complex phage therapies are developed and tested, consideration of the luminal microenvironment—critical to GI health—is required.

One of the most important components of the luminal microenvironment is mucin. Mucins are abundant in the GI tract, and span from the proximal to distal end to protect the underlying mucosa. They are high-molecular weight, highly glycosylated proteins that occur in one of two forms: transmembrane or gel-forming. Transmembrane mucins form a protective brush border on the intestinal epithelium, while secreted mucins form a gel proximal to the epithelium, providing hydration and protection from shear stress [26]. The luminal mucin is a microenvironmental niche with potential to stimulate not only microbiota–host interactions but also inter-microbial interactions. Changes in the synthesis, secretion, and structure of the mucin in both the small intestine and large intestine have been identified in GI disorders, which, in turn, is likely to affect all interactions within the GI environment [27,28,29,30,31,32]. In this review, we examine the interactions between mucin and microbiota to suggest how manipulation of mucin may ultimately influence the effectiveness of phage therapy for complex GI disorders.

## 2. Gastrointestinal Mucin

**Figure 1:**
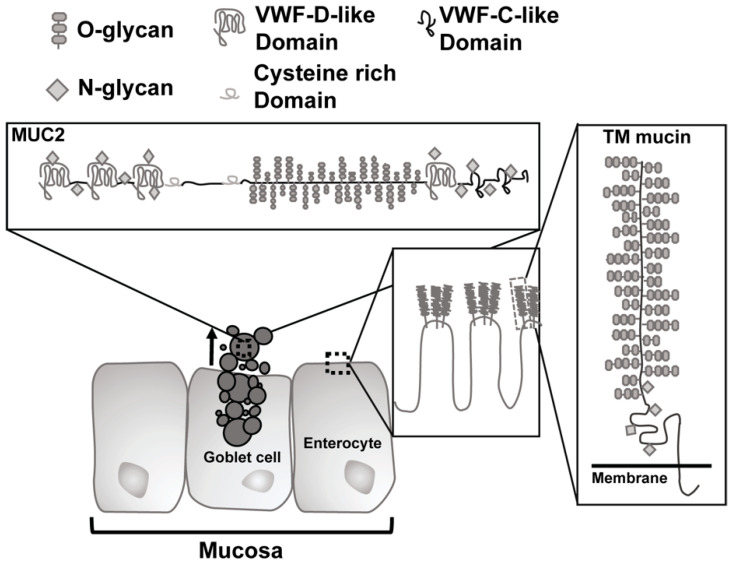
Schematic of types of mucin in the gastrointestinal tract. Secreted mucins, such as Muc2, are generated by goblet cells and form a gel-like covering of the mucosa. Transmembrane ™ mucins create the glycocalyx and are found on the membranes of enterocytes. VWF = von Willebrand factor.

The core proteins of GI mucin contain domains consisting of proline, threonine, and serine (PTS domains) which become O-glycosylated in the Golgi with *N*-acetylgalactosamine (GalNAc) via activity of peptidyl-GalNAc transferases, which regulate glycosylation density [33,34]. The GalNAc residues then serve as substrate for further glycosylation with Galactose (Gal) or *N*-acetylglucosamine (GlcNac). Glycosylation is a dynamic process and there is variation between mucin polymers and along the intestinal tract with an increase in sulfated mucins in the distal regions [33,35]. Mucin expression is distinct to the organs they are located in, with two types of mucins in the GI tract: transmembrane mucins and gel-like mucins [29,30,36,37]. The transmembrane mucins (MUC1, MUC3, MUC4, MUC12, MUC13, MUC16, and MUC17) all possess a cytoplasmic tail and a massive extracellular mucin domain that is highly glycosylated (Figure 1). In the intestine, MUC3, MUC12, MUC13, and MUC17 glycosylated mucin domains form a stiff “brush” protruding from enterocytes to form what is termed the glycocalyx [38].

The glycocalyx remains attached to the epithelium and provides a type of protective barrier for the luminal side of the enterocytes that line the gastrointestinal tract. In contrast, the gel-forming mucins (MUC2, MUC5AC, MUC5B, and MUC6) are secreted from goblet cells in the small and large intestine to coat the gastrointestinal mucosa. They possess cysteine-rich domains in both the N- and C-terminal regions that resemble organization of von Willebrand factor with several von Willebrand D domains [7]. These mucins are extremely large, heavily *O*-glycosylated polymers that are compacted into the granules of the goblet cells in low pH and high calcium conditions [33,39]. Upon secretion, sodium bicarbonate mediates an increase in pH and chelation of calcium ions to allow swelling of packed mucin via their *O*-glycans. Subsequent cleavage by proteases releases the mucin to allow for formation of mucin matrices over the mucosal epithelium [37,40]. Of the gel-forming mucins, only MUC2 is found within the intestine and demonstrates different properties depending on its location. In the small intestine, secreted MUC2 forms long linear polymers via disulfide bonds. In turn, these polymers form a mucus protein scaffold; a single, loosely attached layer that is penetrable by bacteria. Typically, turnover of this layer occurs rapidly with evidence for villi goblet cells replenishing mucin stores within 1–4 h and turnover of secreted mucin occurring in less than a day [30,41]. The mucin sloughs off the surface and the migrating motor complex of the intestine during the fasted motility state moves it toward the colon with all the microbiota contained within it. In addition to the constant turnover of the mucin layer to protect the mucosa from contact with luminal bacteria, the mucin also aids in the formation of an antimicrobial gradient within the intervillous spaces [29]. Non-covalent interaction between adjacent gel-forming mucin polymers is made possible by contact between regions of highly conserved cysteine residues known as the “CYS” domain on neighboring polymers [42]. This interaction leads to aggregation and is a determinant of the permeability of the gel-forming mucus layer to bacteria.

Mucin of the colon is arranged differently as compared to that of the small intestine. Conventionally, colonic mucin was described as consisting of two separate layers: a tightly packed, adherent inner layer impermeable to bacteria (the unstirred layer, <0.5 µm), and a looser outer layer more akin to what is found within the small intestine (Figure 1). Constant secretion of mucin from the colonic goblet cells was believed to move mucin from the inner to the outer layer with proteolytic cleavage at the delineation point between the inner and outer layers, creating a looser, outer form of mucin [34,37,43]. However, a newly proposed model of mucin structure in the colon was recently proposed by Bergstrom et al. [44]. This new model still has two layers, but there are differences in the mucin based on its origin (proximal vs. distal colon), biochemical structure, and function. Proximally, the colonic mucin consists of a mucus “niche” (where bacteria and phages are localized) and a “barrier” layer. The distal colon contains both a proximal colon-derived “b1 layer” situated on top of a distal colon-derived “b2 layer”. Turnover of mucin still occurs in this new model, but mucin encapsulates the bacteria along with fecal pellets, representing mucin-wrapped bacteria, moving material distally for eventual expulsion and providing the b1 layer in the distal colon.

### Function of Mucin

The main function of mucin is to protect the intestinal mucosa from constant and direct physical contact with commensals, as well as from invasion by pathogens. The gelatinous nature of mucin limits the diffusion of bacteria through this space [45,46] confining bacteria–epithelia interactions to the tips of the intestinal villi [33,45]. Its constant turnover in combination with the squeezing effect of intestinal motility towards the large intestine also prevents sustained contact. Additionally, mucin of the small intestine houses an antimicrobial gradient from the crypts outward due to the antimicrobial peptides and proteins secreted by Paneth cells located at the base of the crypts that prevents bacteria from moving beyond the outer edges of the mucin layer [45,47,48]. The glycocalyx layer of the small intestine provides the mucosa with a more direct barrier to bacterial contact, thus protecting enterocytes from the commensals or invading pathogens that may come into proximity. In contrast, within the colon, Bergstrom et al. posited it is encapsulation of bacteria by mucin of the proximal colon that primarily helps to protect the mucosa [44]. A less well-appreciated function of mucin is its role in facilitating the interaction of phages with bacteria, an area that will be discussed in this review.

## 3. Microbial Interactions with Mucin during Homeostasis

### 3.1. Bacteria and Mucin

Bacteria and mucin have a mutualistic relationship. The presence of bacteria upregulates mucin production and enhances their encapsulation by mucin in the colon [44,49,50]. In germ-free mice, the inner layer of colonic mucus is thin, as compared to wildtype mice, and MUC2 is abnormally anchored to the goblet cells in the small intestine [43]. This is due to the lack of bacteria which are necessary for stimulating the release of the enterocyte protease meprin β, the enzyme responsible for cleaving MUC2 from the goblet cells upon secretion [40,43]. In addition to providing a habitat for bacterial populations, mucin also serves as a food source. Evolutionary changes in bacteria have facilitated mucin interactions as many bacterial species carry lectin-type adhesins allowing adherence to mucin glycans [49,51,52] and glycan degrading enzymes for slow degradation of glycosylation residues to serve metabolic needs [53,54]. Bacterial metabolism of the mucin glycans is mutually beneficial, as metabolism byproducts include short chain fatty acids that can diffuse to the mucosa and provide a source of critical fuel for gastrointestinal epithelial cells [29,55].

In the context of phage therapy, it may be possible to exploit the mucin–bacteria mutualistic relationship to improve therapeutic outcomes. By providing the luminal environment with substrates for bacterial metabolism (prebiotics), we can increase the availability of desirable short chain fatty acids, thus externally influencing mucin production. For example, the short chain fatty acid butyrate is well studied as a signal for promoting mucin production [56]. Strategies for increasing butyrate availability to the gut include administering prebiotics such as xylo-oligosaccharides (XOS), fructo-oligosaccharides (FOS), galacto-oligosaccharides (GOS), and inulin fructans that are converted to butyrate by the metabolism of these substrates by butyrate-producing bacteria such as *Faecalibacterim praunsnitzii* (a Clostridium cluster IV strain) [57]. In addition to indirectly increasing mucin production and the luminal availability of butyrate, these substrates provide food source for beneficial butyrate-producing bacteria which have been shown to inhibit *C. dificile* in mice, suggesting the beneficial effects of butyrate on the gastrointestinal microbiota [58]. Alternatively, a strategy exploiting the cross-feeding interaction between Bifidobacterium spp., producing acetate and lactate from the fermentation of starch, and butyrate-producing bacteria that convert these byproducts into butyrate could also be used to increase the availability of this mucin-promoting short chain fatty acid [59]. Administration of pre and/or probiotics offers a method of potentially manipulating the mucin microenvironment towards health in conjunction with phage therapy treatment.

### 3.2. Phage and Mucin

Phages are typically thought to infect a single species or closely related genera of bacteria. However, the recent identification of promiscuous phages able to infect several different bacterial genera was described, suggesting that phage populations may be capable of infecting a much broader repertoire of bacteria [60]. In order to infect bacteria, the phage must first bind. There are a variety of reported mechanisms by which phages may attach to bacteria, including via glycan-binding proteins [61]. Proteins with glycan recognition domains may serve a dual purpose, allowing the phage to attach to the bacteria on which they predate as well as to adhere to glycan modifications in the surrounding environment, including those on mucin. One such example is the immunogenic Hoc protein that decorates the capsid of the phage. Hoc is made up of three immunoglobulin (Ig)-like domains and a fourth non-Ig-like domain [62]. Most Ig-like domains function in recognition and adhesion processes and provide a built-in hypervariability similar in manner to the immunoglobulins to which they are related [63]. Together, these features translate to recognition adaptability for a variety of glycan ligands. Evolutionarily speaking, this variance would help to maintain phage populations within an environment where glycan targets are changed and modified constantly, not only on the surfaces of their target bacteria, but also including modifications on the mucins of the surrounding luminal environment (Table 1). Phage glycan-binding proteins have binding affinities on par with those of most antibodies [61,64], which, in the context of mucin, would allow for retention of phage within the space.

Phages are not the only members of the microbiome to take advantage of the glycan modifications of mucin. As described above, bacteria also interact with mucin, foraging glycan residues as an energy source or anchoring within a region of the gastrointestinal luminal environment [54]. Thus, a mucin-centric interplay between phage and bacteria occurs. In 2013, Barr et al. proposed a new model for phages within the gastrointestinal lumen termed “bacteriophage adhering to mucus” (BAM) [65] supported by previous work from Dabrowska et al. [75]. In this work, a coliphage bound to mucin through the Ig-like domains in its capsid proteins—the T4 Hoc protein. Furthermore, this interaction resulted in decreased numbers of *E. coli* within the same microenvironment in an infection-dependent manner. These data suggest that phage binding to mucin serves to aid in the retention of T4 within the mucin and increase encounter rate between the phage and *E. coli* within the same space [65,66]. Green et al. showed that T4 is not the only coliphage with mucin binding capabilities by identifying another coliphage which binds to heparan sulfated proteoglycans of the glycocalyx and mucus through a tail domain [68]. Importantly, this work demonstrated that not only did mucin binding provide an opportunity for the phage to interact with its bacterial host, but proved necessary for phage-mediated lysis of the bacteria. While these highlighted studies indicate the mucin-centric phage–bacteria interaction in the light of predation, there are other possible interactions that may occur including that of an inhibitory nature. The consideration here is whether the binding of phages to available glycan residues on mucin in turn selectively inhibits the binding of bacteria. Would it be possible for this inhibition to also allow for increased predation as the bacteria move within the mucin microenvironment? Additionally, would shifts in mucin glycosylation—loss or change in modifications—result in correlated shifts in the phage population capable of residing in the mucin? Better understanding of phage–mucin interactions are necessary to answer these questions.

While a substantial number of phages are yet to be tested for their mucin binding ability, there are indications that phage–mucin interactions may be common within the luminal environment simply based on the types of modifications phages have been shown to interact with [66,67,68,73]. There are several reports of phages binding to residues that are consistent with those found on mucin. Specifically, Porayath et al. characterized several coliphagic phages isolated from sewage that were bound to the human matrix molecules fibronectin and heparin [67]. These are proteins that undergo posttranslational modification by glycosylation similar to mucin. Dong et al. found that M13 bound strongly to sulfur when working to engineer a bio-based nanostructure [66]. While this approach was not intended to highlight the ability of this phage to bind mucin molecules, the implication is that M13 might also bind to sulfonated residues on mucin. This property would be particularly interesting as sulfonation of mucin increases as one moves distally along the intestinal tract, suggesting that phages might not only possess an approach for maintaining their populations in mucin but may be able to spatially orient themselves in the intestinal tract. Similarly, the coliphagic phage K1F encodes for an endosialidase in its tail region which binds to and cleaves sialic acid [73]. This particular protein is advantageous to a phage attempting to infect *E. coli* K1 that are encapsulated with polysialic acid. It is then possible that a phage could also recognize sialic acid residues on mucin not only for binding, but also for cleaving, in a similar action to that which it performs on its bacterial host. Given these data, it is likely that most of the phage population within the GI tract is capable of attaching to mucin for retention or predation purposes.

It is also possible that the mucin microenvironment plays a direct role in the ability of phage to infect their corresponding bacteria. Lark and Adams examined the effects of mono- and divalent cations on T2, T4, and T5 phage and suggested that the ionic environment in which phage reside affected virion substructure such that ion concentration could prove detrimental or protective [76]. In 2017, Szermer-Olearnik et al. built on this work characterizing the physiological effects of sodium monovalent cation on purified T4 phage cultures [77]. Herein, they described an aggregative effect on T4 phage when sodium concentrations were dropped from 150 to 10 mM with pH serving as a modifier of aggregation kinetics. These types of phage aggregates have been observed naturally and are thought to assist in phage stabilization [78]. Although these experiments were carried out with monovalent ions to avoid the enzymatic properties associated with divalent cations, it is likely that divalent cations can effectuate similar aggregation with certain phage. As both calcium and pH are essential during the secretion of gel-like mucins, as well as in the formation of an intact mucin layer, phage interactions with the surrounding mucin may vary during fluctuations in either.

## 4. Dysfunction of Mucin

### 4.1. Small Intestine

The role of mucin dysfunction in colonic disease is well studied. Useful insights into the effects of perturbation of the synthesis/secretion of mucin within the small intestine can be gleaned from studies on cystic fibrosis (CF) and CD. Cystic fibrosis is a mucin-centric disease where patients characteristically suffer from excessive production of abnormal mucus in their lungs. However, the disease also affects the mucus in the GI tract. As treatments have improved and lifespans of CF patients have increased, there has been a concomitant rise in the number of patients with GI mucin complications of the small intestine including distal intestinal obstruction syndrome and small intestinal bacterial overgrowth (SIBO) [29]. In the intestine, the cystic fibrosis transmembrane conductance regulator (CFTR) chloride channel resides in the apical surface of enterocytes and mediates secretion of chloride and bicarbonate across the mucosal layer [79]. Along with other intestinal ion channels and transporters, CFTR plays a crucial role in maintenance of the salt and water flux in the lumen to maintain pH and fluidity of the luminal environment. In CF patients, CFTR does not function properly, is not produced at high enough levels, or is missing completely. So, in the intestines of these patients loss of CFTR function results in changes in luminal pH and fluidity which, in turn, result in dysfunction of the mucin pathway; altered biophysical properties of mucin so that secreted mucin is tightly compacted rather than swelling and expanding to loosely cover the intestinal mucosa [29,80]. Because the mucin is now firmly adhered to the mucosa, a significant amount of luminal material (i.e., microbiota and food particles) becomes entrapped and no longer moves distally toward the colon with peristalsis. Mucin adherent bacteria are retained in this space, accumulate, proliferate, and expand spatially in an exponential fashion leading to bacterial overgrowth, which in turn triggers a negative feedback loop for increased mucin secretion, compounding the problem. Even normal flow through the small intestine may be obstructed as a consequence. Within the retained microbiota, there is also a shift in the bacterial constituents that correlates to disease severity. Bacterial changes include: loss of diversity, increased abundance in Firmicutes with concurrent loss in abundance of Bacteroides, and increased abundance in Enterobacter and Bifidobacterium [80,81].

Crohn’s disease (CD) of the small intestine occurs most commonly in the terminal ileum but can affect any part of the intestinal tract and is characterized by zones of deeply inflamed tissue that might be adjacent to non-inflamed tissue. In CD, mucin dysfunction is related to stress occurring along many of the steps of the mucin synthetic pathway. As mucins are large (megadalton), heavily glycosylated proteins, they require sufficient time for appropriate processing prior to secretion. During the disease, signaling for increased mucin secretion reduces time available for proper processing resulting in misfolding, decreased levels of glycosylation, and subsequent change to mucin conformational structure and its protective capabilities [33,82]. Although mucin in CD patients is still secreted, the relationship between CD and mucus remains in dispute, with reports of both increases and decreases in mucus thickness [83]. A meta-analysis of mucin expression in CD found a 34% reduction in total mucin due to decreased levels of MUC5AC, MUC5B, and MUC7 [84]. A decrease in MUC2 expression also occurs and mucins not typically expressed in the ileum have also been found in CD patients. The mucin that is expressed in CD have shorter glycan residues and lower rates of sulfonation. Without the typical amounts of glycosylation, bacteria-binding sites may be lost setting the stage for shifting the bacterial species capable of retention within the luminal environment and providing opportunity for expansion of pathobionts. The bacterial metabolism of mucin also changes when dysfunction in mucin synthesis occurs. Typically, bacteria metabolize mucin one glycosylated residue at a time, allowing for the core to remain intact. Loss of extensive glycosylation results in more rapid and more complete mucin breakdown leaving larger “holes” in the mucin matrix that would typically protect the mucosa.

### 4.2. Large Intestine

In the colon, changes in mucin also result in the triggering of disease states, as can be seen in ulcerative colitis (UC). Inflammation characteristic of UC is superficial and radiates proximally from the rectum with a thin, discontinuous mucus layer covering the mucosa [83]. UC patients have decreased numbers of goblet cells and have been found to express MUC5AC, a mucin that is not typically expressed in the colonic environment but plays a critical role in protecting the mucosa in this diseased condition by blocking the access of bacteria to the mucosa [85]. In addition to being depleted in numbers, the goblet cells in diseased tissue appear unable to keep up with mucin replenishment so as to exhaust their mucin supply. Among other observed mucin-related changes during active UC, patients have a decreased expression profile of MUC2 and the mucins that are secreted have reduced glycosylation and sulfonation, and increased sialylation. These changes in mucin structure allow for more bacterial enzymatic degradation and permeabilization of the mucin barrier to the luminal bacterial population. In inflammatory diseases of both the small and large intestine, mucin is a critical component in the interaction between bacteria and mucosal environment, and its dysfunction can drive changes in the bacterial composition while increasing the allowed exposure of the mucosa to bacteria and bacterial byproducts, all of which could be detrimental to the body.

### 4.3. Mucin-Mediated Phage Interactions

While data suggest dysfunction of mucin in dysbiosis or GI disease influences bacteria–host interactions, phage interactions with both bacterial and metazoan hosts are also likely to be influenced in a meaningful way, as it has been shown that both bacterial and phage populations shift dramatically in a number of GI disorders [86,87,88]. In a healthy GI environment, the majority of the phage population is thought to exist in a lysogenic state [6,89,90]. However, in a dysbiotic GI tract, lytic phages become more prevalent and certain phage populations expand exponentially [89,91]. While stress on, or lysis of, particular bacterial hosts may be responsible for the expansion of these phage populations, it is also possible that changes in the mucin contribute to these shifts. For example, decreased glycosylation of mucin that occurs due to ramped up synthesis may remove recognition sites for both phage and bacteria, thereby decreasing the likelihood that predation could successfully occur. Reducing the probability of successful predation could lead to a concurrent depletion of selected phage populations and result in blooms of pathobionts (such as sulfur reducing bacteria [92,93,94]) that are normally tightly controlled to keep population numbers low. Alternatively, an increased number of phages in the luminal environment could saturate glycan binding sites within the mucin thus “locking out” beneficial bacteria that would normally reside there, allowing them to be flushed out of the intestinal tract. In this scenario, loss of beneficial bacteria would remove competition for resources, thus allowing for pathobiont bloom. It is also possible that the loss of glycan residues may eliminate food sources for certain bacteria, allowing for increases in populations of pathobionts, such that phage predation is insufficient to control numbers. If stressors on certain bacterial populations result in a loss of mucin or abnormal mucin structure, the binding sites on mucin may become unavailable to facilitate phage predation. Again, blooms in pathobionts, as seen associated with a variety of GI disorders, may be the result. Finally, if mucin coating of bacteria does not occur as it would in a healthy GI tract, bacteria, previously controlled by mucin-mediated phage interactions, may expand unchecked.

In the event of mucin dysfunction, structural changes of mucin molecules could represent the loss or gain of contact points where phage interactions could occur, thus shifting from the native state to an abnormal state. For example, increased sialylation of colonic mucins in UC may result in increased absorption of a particular type of phage, removing it from the milieu and decreasing the availability of a predator of its bacterial counterpart. Alternatively, expression of mucins such as MUC5AC in the colon during the disease may also introduce novel phage binding sites thereby impacting the normal phage dynamics and, indirectly, affecting the bacterial population. With all of these possibilities comes the realization that mucin-mediated phage–bacteria–mucosa interactions are not well understood, leaving this field of research ripe for investigation.

## 5. Modifying Mucin to Facilitate Phage Therapy

In instances of GI dysbiosis where mucin dysfunction occurs, how can we utilize mucin-mediated interactions to our advantage in combination with phage therapy? While conventional phage therapy has been successful against specific pathogens, including those invading the GI tract [2,7,8], there has been less success in treating complex GI disorders. The idea of modifying phage therapy to match the complexity of the bacterial dysbiosis by using fecal virome transfer that would present a repertory of predators makes sense and has been useful to some degree [17,87,95]. Is manipulating the mucin microenvironment in conjunction with phage therapy to shift the gastrointestine toward health possible? While we do not currently know the answers to these questions, it is undeniable that given the critical role of mucin not only in maintenance of the GI tract and in mediating phage-bacteria-host interactions, its state and available quantity prior to and during phage therapy may need to be considered to ensure maximal therapeutic benefit. These considerations might include approaches that increase or reduce the availability of mucin or modify the overall structure of mucin polymer or how they aggregate together to form a scaffold that facilitates phage-bacteria interaction. These strategies could be accomplished by using prebiotics or probiotics to enhance the luminal environment in a desirable manner. As gel-forming mucin is made up of >90% water [42], strategies directed at changing the hydration of mucin is likely to modulate phage–bacteria interaction. Additionally, mucin-active proteases or compounds to regulate pH and/or salt concentrations could be applied before or during phage therapy treatments. Regardless, a better understanding of phage–mucin interactions is necessary in order to optimize these relationships for therapeutic benefit.

## 6. Summary

As the mucin regulates several luminal interactions critical to phage–bacteria predation, it is important to better understand the overall state of mucin within a dysbiotic system. This is particularly important for phage therapy of complex GI disorders without the use of antibiotics. Consideration of the dynamics influenced by mucin synthesis, secretion, and structure during phage therapy may illuminate ways to make treatment more effective, including the generation of mixed phage cocktails with phage(s) selected specifically with the mucin microenvironment in mind. Alternatively, inclusion of probiotics or prebiotics to support “healthy” mucin during phage application may help to ameliorate disease, thus targeting mucin as adjunct to phage therapy for complex GI disorders.

## Figures and Tables

**Table 1:** Reported phage interactions with mucin and mucin-like domains.

Phage (Bacterial Host)	Phage Protein Involved in Interaction	Mucin Residue Involved in Interaction	Demonstrated ^1^	Reference
*Ligand Interactions*				
T4 (*Eschericia*)	Hoc	unknown	known	[65]
M13 (*Eschericia*)	P8	Sulfonation site	suggested	[66]
Coliphage (*Eschericia*)	unknown	glycans	suggested	[67]
ES17 (*Eschericia*)	unknown	unknown	known	[68]
T5 (*Eschericia*)	unknown	oligomannose	suggested	[69]
PlyP35 (*Listeria*)	CBDP35	GlcNAc	suggested	[70]
ϕ11 (*Staphylococcus*)	Gp45	GlcNAc	suggested	[71]
ϕ187 (*Staphylococcus*)	unknown	GalNAc	Suggested	[72]
*Enzymatic Interactions*				
K1F (*Eschericia*)	endosialidase	Sialylation site	suggested	[73]
ϕ1.2 (*Eschericia*)	endosialidase	NeuNAc	suggested	[74]
ϕ 92	endosialidase	NeuNAc	suggested	[74]

^1^ Known = demonstrated interaction between phage and mucin; Suggested = demonstrated interaction between phage and mucin-like domain.

## Data Availability

No new data were created or analyzed in this study. Data sharing is not applicable to this article.
